# Antihypertensive Effect of a Novel Angiotensin II Receptor Blocker Fluorophenyl Benzimidazole: Contribution of cGMP, Voltage-dependent Calcium Channels, and BK_Ca_ Channels to Vasorelaxant Mechanisms

**DOI:** 10.3389/fphar.2021.611109

**Published:** 2021-03-30

**Authors:** Hina Iqbal, Amit Kumar Verma, Pankaj Yadav, Sarfaraz Alam, Mohammad Shafiq, Divya Mishra, Feroz Khan, Kashif Hanif, Arvind Singh Negi, Debabrata Chanda

**Affiliations:** ^1^ Bioprospection and Product Development Division, CSIR-Central Institute of Medicinal and Aromatic Plants, Lucknow, India; ^2^ Phytochemistry Division, CSIR-Central Institute of Medicinal and Aromatic Plants, Lucknow, India; ^3^ Computational Biology Lab, CSIR-Central Institute of Medicinal and Aromatic Plants, Lucknow, India; ^4^ Division of Pharmacology, CSIR-Central Drug Research Institute, Lucknow, India

**Keywords:** hypertension, benzimidazole, BK_Ca_ channel, l-type VDCC, SHRs, cGMP

## Abstract

**Background:** The current study presents the novel angiotensin II receptor blocker fluorophenyl benzimidazole (FPD) as an antihypertensive agent in the SHR model of hypertension. We investigated the role of cGMP, voltage-dependent L-type calcium channels, and BK_Ca_ channels in the vasorelaxant mechanisms of FPD in the rat superior mesenteric artery.

**Methods:** The antihypertensive effect of FPD was examined using an invasive technique measuring blood pressure in SHR animals. Using a myograph, tension measurement was completed in the superior mesenteric artery to elucidate the mechanisms of vasorelaxation involving AT1 receptors, the NO/cGMP pathway, L-type calcium channels, and BK_Ca_ channels. Ion flux (Ca^2+^, K^+^) studies were conducted in aortic smooth muscle cells. Putative targets proteins were determined by *in silico* docking studies. A safety evaluation of FPD was carried out using Swiss albino mice.

**Results:** FPD significantly decreased blood pressure in SHR. It relaxed superior mesenteric arteries in a concentration-dependent manner and significantly inhibited angiotensin II-induced contraction. The relaxation response was also mediated by an increase in tissue cGMP levels, inhibition of L-type calcium channels, and the opening of BK_Ca_ channels. FPD further enhanced efflux of K^+^ and inhibited Bay K8644-stimulated Ca^2+^ influx in aortic smooth muscle cells and docked well in an *in silico* study with the targets. It was well tolerated in the toxicity study.

**Conclusion:** The present study reports the antihypertensive activity of novel AT-1 receptor blocker FPD at 50 and 100 mg kg^−1^ with cGMP, L-type calcium channels, and BK_Ca_ channels as putative targets of vasorelaxation, and was found safe in oral toxicity.

## Introduction

Non-communicable diseases are the leading cause of death in the developed and developing world. They are responsible for approximately 68% of all global deaths, out of which approximately 46.2% of deaths occur due to cardiovascular diseases worldwide (WHO, 2017). Benzimidazoles and substituted benzimidazoles have been used for various biological activities including hypertension since 1944 (Woolley, 1944). Over time and through active research interest, benzimidazoles have emerged as an important heterocyclic class of molecules due to their clinical use as drugs like omeprazole as an antiulcer treatment, albendazole as an antiparasitic, benoxaprofen as an anti-inflammatory, bendamutine as an antineoplastic treatment, astemizole as an antihistamine, and pimobendane as a calcium sensitizer, etc. (Supplementary Figure S1A) (Bansal and Silakari, 2012). Further, benzimidazole has often been used as an important basic core, especially for the development of antihypertensive agents. Telmisartan, candesartan, and azilsartan have emerged from this class of compounds as clinical drugs acting through the angiotensin receptor blockade (AT1). In spite of the vast number of studies on benzimidazoles and angiotensin receptor blockers (ARBs), literature on the role of these classes of compounds on VDCC and potassium channel functioning pertaining to vasorelaxation in conduit and resistance arteries and antihypertensive activity has been very limited. In the present study, we planned to create a phenyl ring at the C2 position of a benzimidazole core, instead of N-benzylbiphenyl as in candesartan and telmisartan, to obtain a small molecular weight pharmacophore. A fluorine group was also kept to create a high electron density for better interaction, similar to a H-bond acceptor, with the receptor protein. All the 2-arylbenzimidazole derivatives were evaluated for vasorelaxation and the most potent molecule, fluorophenyl benzimidazole was studied for antihypertensive activity and elucidation of its mechanism of action through blocking the AT1 receptor, cGMP buildup, and modulation of the BK_Ca_ channel and VDCC function.

## Materials and Methods

### Chemical Synthesis

Synthesis of 2-(4′-Fluorophenyl)-1H-benzimidazole (34/FPD): o-Phenylenediamine (128 mg, 1.2 mmol) and 4-fluorobenzaldehyde (124 mg, 1.0 mmol) were taken in dry methanol (10 ml). To this reaction mixture, 1.0 g of molecular sieves (3Å) was added and the reaction mixture was refluxed for 1 h (Supplementary Figure S1B). On completion, the reaction mixture was filtered and solvent was evaporated *in vacuo*. The residue was purified through column chromatography over silica gel (60–120 mesh), eluted with chloroform-acetone (99:1), and recrystallized from hexane-chloroform to get benzimidazole 34 as creamy amorphous powder in 89% yield.

Yield = 89%; creamy-amorphous powder; M.P. = 233–235°C; ^1^H NMR (CD_3_OD, 500 MHz): δ7.19–7.22 (m, 4H, 4xCH, aromatic), 7.58–7.56 (d, 2H, 2xCH, aromatic), 8.04–8.06 (m, 2H, 2xCH, aromatic); ^13^C NMR (CD_3_OD, 125 MHz): δ117.08, 117.25, 124.08, 127.58, 130.10, 130.16, 152.53, 164.47, 166.46; ESI-TOF HRMS (MeOH): m/z [M+H]^+^ calculated for C_13_H_10_FN_2_, 213.0828, found 213.0823. Physical data of the rest of the compounds were recently reported (Chaturvedi et al., 2018).

### Drugs

U46619 (a thromboxane analogue), acetylcholine (acetylcholine chloride; ACh), dimethylsulfoxide (DMSO), 1H-[1,2,4]oxadiazolo [4,3-a]quinoxalin-1-one (ODQ), trichloroacetic acid (TCA), calcium chloride (CaCl_2_), EGTA, Bay K8644, tetraethyl ammonium (TEA), iberiotoxin, 4-aminopyridine (4-AP), glibenclamide, angiotensin II, smooth muscle α-actin antibody, TRITC-phalloidin, and 4′6-diamidino-2-phenyl indole (DAPI) were obtained from Sigma-Aldrich (St Louis, MO-United States). Papain (EC number: 3.4.22.2), collagenase (EC number: 3.4.24.3), trypsin (EC number: 3.4.21.4), and DMEM were purchased from Sigma Chemicals, India Limited. All other chemicals and salts were purchased from E-Merck India Limited and Sigma Chemicals, India Limited. A cGMP (cyclic guanosine monophosphate) ELISA kit was obtained from Cayman Chemical (Anna Abor, MI, United States). Fetal Bovine Serum (FBS), a FluxOR™ II Green Potassium Ion Channel Assay kit, and Fluo-4 NW Calcium Assay Kits were obtained from Invitrogen (Paisley, United Kingdom). The stock solutions of the molecules studied were prepared in DMSO and were further diluted in bath fluids. The final concentration of DMSO did not exceed 0.1% in the bath solution.

### Animals

All animal care and experiments were carried out following the Institutional Animal Ethics Committee (IAEC) approved protocols (CIMAP/IAEC/2016(07)-2019/33, CIMAP/IAEC/2016-2019/01 for hypertension and toxicity studies, respectively) followed by recommendations of the Committee for the Purpose of Control and Supervision of Experiments on Animals (CPCSEA), Government of India. All the animals were maintained with an automated dark/light cycle of 12 h with controlled temperature (22 ± 5°C) and humidity (45 ± 5%). The animals were fed with the standard rat feed and were provided with drinking water *ad libitum*. Prior to the actual experiments, all the animals were acclimatized for 7 days in the experimental environment. For the *ex vivo* and *in vitro* studies, male and female Wistar rats of 180–250 g were used, while male spontaneously hypertensive rats (SHRs) were used to assess *in vivo* antihypertensive activity. Male and female Swiss albino mice were used in acute and sub-acute oral toxicity tests.

### Tissue Preparation for *Ex Vivo* Vasorelaxation Study

Wistar rats weighing 180–250 g were sacrificed under anesthetized conditions using ketamine (80 mg kg^−1^) and xylazine (10 mg kg^−1^). The intestine was excised and placed in aerated cold modified Krebs-Henseleit solution (MKHS) containing 118 mM of NaCl; 24 mM of NaHCO_3_; 4 mM of KCl; 1.8 mM of CaCl_2_; 1 mM of MgSO_4_; 0.43 mM of NaH_2_PO_4_; and 5.56 mM of glucose. The superior mesenteric artery was dissected and cleaned from the surrounding adipose tissue, and cut into rings of 2–3 mm in length. These rings were mounted in a dual-chamber small-vessel wire myograph (Danish Myo Technology, Aarhus, Denmark) containing 5 ml of MKHS maintained at 37°C with continuous bubbling in carbogen gas (95% oxygen and 5% carbon dioxide). The tissue was stretched to the basal tension of 5–6 mN and equilibrated for 60 min with continuous change in buffer at every 15 min. The tissue viability was assessed by high potassium depolarizing solution (80 mM) evoked contraction, and endothelium integrity was confirmed by ACh relaxation response over U46619 (100 nM)-induced contraction and was used for the assessment of vasorelaxation response of benzimidazole analogues (Singh et al., 2019).

### *In vivo* Antihypertensive Activity in SHRs

Six to eight-week-old male SHRs weighing 200–250 g were taken for the study. FPD was solubilized in groundnut oil and was administered orally to SHRs at a dose of 50 mg kg^−1^ and 100 mg kg^−1^ while the vehicle control group received only groundnut oil. A known antihypertensive drug, minoxidil (a potassium channel opener), was dissolved in water and was given orally at a dose of 10 mg kg^−1^. All the animals were subjected to the treatment for 14 days. After 14 days, the hemodynamic parameters and heart rate of all the animals was studied using an invasive blood pressure method. Invasive blood pressure measurement was done by anesthetizing the animal with ketamine (80 mg kg^−1^) and xylazine (10 mg kg^−1^). The carotid artery was exposed, a non-occlusive polyvinyl catheter (PE catheter) was inserted, and blood pressure was measured using an analog-to-digital interface (PowerLab Data acquisition system, ADInstruments Inc., Australia). The data ware analyzed by LabChart software V 8.0.

### Elucidation of Mechanisms of Vasorelaxation: Ion Channels and Pathways

To elucidate the role of the endothelium in the relaxation response, vascular reactivity was studied in both intact and denuded endothelium rings. Denudation of the endothelium was done mechanically by rubbing the lumen of blood vessels with human hair and was confirmed by loss of relaxation response to acetylcholine (10 µM) to less than 10%. Since, most of the antihypertensive benzimidazoles are known to act via blockade of the AT1 receptor, we checked the most potent molecule FPD (c-substituted fluorophenyl benzimidazole) for AT1 receptor blocking activity by pre-exposing mesenteric arterial rings to FPD (10 µM) for 20 min followed by elicitation of contraction response to Ang II (1 µM) (Jain et al., 2020).

The contribution of potassium channels in the relaxation response was explored by eliciting contraction response with both 80 mM of high K^+^ MKHS (containing 42.7 mM of NaCl; 24 mM of NaHCO_3_; 80 mM of KCl; 1.8 mM of CaCl_2_; 1 mM of MgSO_4_; 0.43 mM of NaH_2_PO_4_; and 5.56 mM of glucose) and a low potassium depolarizing solution (25 mM of high K^+^ MKHS containing 97.7 mM of NaCl; 24 mM of NaHCO_3_; 25 mM of KCl; 1.8 mM of CaCl_2_; 1 mM of MgSO_4_; 0.43 mM of NaH_2_PO_4_; and 5.56 mM of glucose), prepared by equimolar replacement of NaCl with KCl in MKHS. The cumulative concentration-response to FPD was studied. Further, to identify the involvement of potassium channel subtypes in the relaxation response of FPD, superior mesenteric arterial rings were pretreated with blockers of BK_Ca_ channel, TEA (1 mM) or iberiotoxin (10 nM); K_ir_ channel (BaCl_2,_ 30 µM); K_V_ channel (4-AP, 300 µM) and K_ATP_ channel (glibenclamide, 10 µM), and the relaxation response to FPD was elicited in a concentration-dependent manner in agonist pre-constricted arterial rings. U46619 (100 nM) was used as an agonist for inducing contraction for all the potassium channel subtype blockers except glibenclamide where noradrenalin (10 µM) was used as an agonist for pre-incubation because glibenclamide markedly inhibits contraction response to U46619 (Chanda et al., 2016).

To evaluate the role of the sGC-cGMP-PKG pathway, the arterial rings were incubated in ODQ (10 μM, a sGC inhibitor) for 20 min and the relaxation response was studied. In another set of experiments, the role of VDCC in FPD-induced vasorelaxation was investigated. Briefly, after checking the tissue viability and endothelium integrity, the involvement of VDCC in FPD-induced relaxation was assessed by a developing concentration-dependent contraction response to Bay K8644 (a known opener of L-type VDCC) in slightly depolarized (High K^+^, 15 mM) tissues in the presence and absence of FPD (Alonso et al., 1989).

#### Measurement of Intracellular cGMP Level

Endothelium-intact rat superior mesenteric artery segments of approximately 5 mg in weight were prepared and kept under physiological conditions in 1 ml of MKHS at 37°C and were aerated continuously with carbogen gas throughout the experimental procedure. After 30 min of equilibration, treatment with compounds was given as follows.

- Vehicle control, DMSO (3 µl for 10 min).
- FPD (10 µM for 20 min).
- FPD (30 µM for 20 min).
- U46619 (100 nM for 10 min).
- U46619 (100 nM for 10 min) followed by FPD (10 µM for 20 min).
- SNP (1 µM for 20 min).

Treated tissues were immediately frozen with liquid nitrogen and were pulverized in pre-chilled pestle and mortar. Two hundred microliters of 5% TCA was added and the samples were homogenized. These samples were centrifuged at 1,500 g for 10 min at 4°C. The obtained supernatant was made free of TCA by extraction with water saturated ether five times, and at the end the residual ether was removed by heating the supernatant at 70°C for 15 min and was used as a source of cGMP. The ELISA was performed using the manufacturer’s guidelines (Cayman Chemical Company, Ann Arbor, United States). Remaining tissue pellet was used for the estimation of protein concentration. For this, the pellet was dissolved in 100 µl of 0.1 N NaOH and the concentration of protein in the pellet was determined using a Pierce™ BCA Protein Assay kit (Thermo Scientific, Rockford, United States) and the level of cGMP was expressed as pmol mg^−1^ protein (Singh et al., 2017).

#### *In vitro* Assessment of FPD-Induced Modulation of Potassium and Calcium Channel Functions in VSMCs

##### Isolation of Primary Rat Vascular Smooth Muscle Cells

The aorta of Wistar rats were aseptically isolated and cleaned of adhering fatty and connective tissue in the presence of 1% antibiotic solution in MKHS. The tissue was washed, chopped, and digested in an enzymatic mixture of papain (1 mg ml^−1^), collagenase (2 mg ml^−1^), and dithiothriotol (1 µM) in HEPES buffer for 30 min at 37°C in water bath. Over the incubation period, the mixture was shaken repeatedly every 10 min. The digested tissue sample was washed three times with HEPES buffer, suspended in DMEM supplemented with 10% FBS. The single cell suspension was seeded in a 25 cm^2^ cell culture flask and was incubated in 5% CO_2_ at 37°C. Rat aortic VSMCs were allowed to grow at 37°C in a 5% CO_2_ incubator. The cells were maintained in DMEM supplemented with 10% FBS, and the medium was replaced twice a week. VSMCs between passages 5 and 9 were used in all the experiments (Prieto-Lloret et al., 2018).

##### Immunocytochemistry of Rat VSMCs

To exemplify and evaluate the purity of the obtained cell populations, immunocytochemistry of rat aortic VSMC was performed following the methodology published earlier (Weber et al., 2011). Briefly, the aortic cell suspension was plated onto the chamber slide and was allowed to attach. The cells were then fixed by 4% paraformaldehyde and 4% polyethelene glycol 600 with 3 mM of EGTA and 2 mM of MgCl_2_ in PBS. Cells were permeabilized with 0.1% Triton X-100 and were washed three times with PBS. The cells were blocked with 5% skimmed milk for 30 min and then were treated with a smooth muscle α-actin antibody (1:500 in 5% skimmed milk) for 60 min. After being washed twice with PBS, the cells were incubated in goat anti mouse IgG (H + L) and DyLight™ 488 conjugated secondary antibody (1:2,000 in 5% skimmed milk) for 60 min in dark. For the visualization of F-actin, the cells were incubated with 0.5 µM of TRITC-phalloidin for 20 min at room temperature. The cell nucleus was stained with DAPI, washed and mounted in the mowiol mounting medium. Cells were examined using a ZEISS Confocal laser scanning microscope (LSM 880 Carl Ziess microscopy, Germany).

##### Potassium Channel Function Assay in VSMCs

To explore the effect of FPD on potassium channel opening, a FluxOR™ II Green Potassium Ion Channel Assay (Invitrogen, Paisley, United Kingdom) was carried out in VSMCs following the manufacturer’s guidelines. In brief, rat aortic smooth muscle cells were resuspended in the growth medium and were plated at 2 × 10^3^ cells per well in a 96-well plate. Cells were allowed to adhere overnight at 37°C. When the cells were attached, growth medium was replaced by the 1X loading buffer. Cells were incubated at 37°C for 60 min to facilitate dye entry. Further loading buffer was removed and assay buffer was added in each well. Treatment with 10 µM of FPD, 30 µM of FPD, 10 mM of TEA, and 80 mM of KCl were given for 20 min in different wells, and the FluxOR II assay was performed using Spectramax i3 (Molecular Devices, United States). The instrument was set at the excitation wavelength of 480 nm and the emission wavelength of 535 nm. Initially, 50 baseline readings were taken and after that 2 mM of thalium ion solution was injected and the fluorescence was studied for 5 min at every 0.4 s. The data were presented in the form of area under curve and a line diagram was plotted as relative fluorescence unit vs. time in seconds.

##### Calcium Imaging

Rat aortic smooth muscle cells were plated at about 30,000 cells per well in a 96-well plate in DMEM with 10% FBS and was left overnight to reach confluency. The rest of the procedure was done according to the manufacturer’s recommendations (Fluo-4 NW Calcium Assay Kit, Invitrogen, Paisley, United Kingdom). Growth medium was removed from the adherent cell cultures and the dye loading solution was added to each well. Cells were allowed to load the dye for 30 min at 37°C. The cells were then exposed to treatment with 10 and 30 µM of FPD for 20 min. Initial baseline fluorescence was measured and thereafter either NE (10 µM) or Bay K8644 (1 µM) was injected both in the presence and absence of FPD and the change in the relative fluorescence was studied to quantify the intracellular calcium concentration. The data were presented in the form of area under curve and line diagram.

### *In silico* Docking Study

To complement the pharmacological observations, the docking studies were done with the identified protein targets. Based on the *ex vivo* observations, we chose angiotensin II receptor 1 (AT1), sGC, BK_Ca_ channels, and L-type VDCC as target proteins and FPD as a candidate molecule. Along with this, we took Bay 41-2,272 (Pubchem CID: 9798973) for sGC; tetraethylammonium chloride (Pubchem CID: 5946) for BK_Ca_ channel, nifedipine (Pubchem CID: 4485) for VDCC, and candesartan (Pubchem CID: 2541) for AT1 receptor as standards for the docking experiment.

#### Homology Modeling

Prior to the docking studies, the mining of the PDB structure of all the targets was carried out and the crystal structures of BK_Ca_ channels (PDB ID: 3NAF), VDCC (PDB ID: 1T0H), and angiotensin II receptor 1 (PDB ID: 4YAY) were considered for docking. Since suitable crystal structures of the target sGC β subunit could not be identified in the protein database, we created the homology model using the SWISS-MODEL Workspace webserver (Alam et al., 2020). The amino acid sequence of sGC (Organism: *Rattus norvegicus*) with the prescribed length was retrieved from the UniProtKB database (P20595). To identify a suitable template protein 3D structure for modeling, we searched the PDB database by BLAST. The model was built based on template protein for human guanylate cyclase soluble subunit beta-1 (PDB ID: 6JT0). The homology 3D protein structure of this protein described as homology model_1 is represented in Supplementary Figure S3.

#### Protein Preparation and Docking With Ligands

The protein preparation protocol of Discovery Studio was used to perform tasks such as inserting missing atoms in incomplete residues, deleting alternate conformations (disorder), removing waters, standardizing the names of the atoms, modeling missing loop regions, and protonating titratable residues using predicted pKs (negative logarithmic measure of the acid dissociation constant). CHARMM (Chemistry at HARvard Macromolecular Mechanics; Cambridge, MA, United States) was used for protein preparation. Hydrogen atoms were added before the processing. Protein coordinates from the crystal structure of the targets were considered for the docking experiment (Alam and Khan, 2018). For the docking and visualization studies, the LibDock program of Discovery Studio version 3.5 (Accelrys, United States, 2013) was used (Alam and Khan, 2019) where protein site features were referred to as hot spots and were of two types (polar and non-polar). The ligand poses were then placed into this polar and non-polar receptor interactions site. For energy minimization, the SMART minimizer algorithm was used. The CHARMM force field was used in the parameterization step. The best conformation of each ligand was selected according to their position in the active site and the docking score. Selected conformations were used for the analysis of interactions with residues of the active site of the targets (Das et al., 2020).

### Toxicity

Acute and sub-acute oral toxicity was carried out following previously published protocol (Chanda et al., 2009; Singh et al., 2019). Swiss albino mice were treated with FPD at 5, 50, 300, and 1,000 mg kg^−1^ once orally in the acute oral toxicity test and at 0.1, 1, 10, and 100 mg kg^−1^ once daily by oral route for 28 days in sub-acute oral toxicity test. The animals were kept under observation during the course of experiment for any sign of illness, mortality, morbidity, and change in way of walking and posture. Body weight was taken, hematology, serum biochemistry, gross observation of vital organs, and their absolute and relative weights were recorded at the end of the acute and sub-acute toxicity experiments.

### Statistical Analysis

Relaxation responses of the benzimidazole analogues were expressed as the percent reversal of the U46619 or high K^+^ MKHS-induced contraction of the rat mesenteric arterial rings. Concentration-response curves were fitted to sigmoidal curves by nonlinear regression analysis (log agonist vs. normalized response, with variable slope), using Graph Pad Prism version 5.00 (San Diego, California, United States) to calculate the pD2 values (log molar concentration of the agonist producing 50% of the maximal response). Concentration-response curves were analyzed with repeated measures two-way ANOVA (treatment vs. concentration), and Bonferroni post tests were used to compare replicate means. The E_max_, AUC values, and *in vivo* comparisons between control and treated groups were made using one-way ANOVA with post hoc analysis using Tukey’s multiple comparison test or *t* test, as appropriate. AUC was also calculated by the GraphPad software. A value of *p* < 0.05 was considered statistically significant. Column statistics were used to calculate mean and standard error and the results were expressed as mean ± standard error of the mean (SEM), with n equal to the number of arterial rings from different animals (biological replicates) or independent experiments.

## Results

### Synthesis of Benzimidazole Analogues

The synthesis protocol is depicted in Supplementary Figure S1B. *O*-phenylenediamine was condensed with various benzaldehydes in the presence of 3Ǻ molecular sieves. Two series of products were obtained on the benzimidazole core, *i.e.*, 2-arylbenzimidazoles (33–48) in 30–89% as major products and 1, 2-diarylbenzimidazoles (49–53) as a minor product (<8%) (Chaturvedi et al., 2018).

### Novel Benzimidazole as Vasodilator in Superior Rat Mesenteric Arteries

All the compounds chosen for the study relaxed the U46199 (100 nM) pre-contracted rat superior mesenteric artery at 10 µM considering acetylcholine-induced (10 µM) vasorelaxation as standard (Table 1). A further three compounds showing E_max_ > 70% were chosen for the concentration-dependent relaxation (Supplementary Figure S2). Out of the molecules studied, FPD produced the best concentration-dependent relaxation response and was taken for the detailed study (Supplementary Figure S2).

**TABLE 1:** Screening of novel benzimidazole class of compounds for vasorelaxation in isolated endothelium-intact rat superior mesenteric arterial rings against U46619 (100 nM)-induced contraction. Acetylcholine (10 µM)-induced relaxation is considered as positive control for the screening (mean ± SEM, *n* = 5).

	Compound (10 µM)	Structure of compound	Percent relaxation
1	FPD	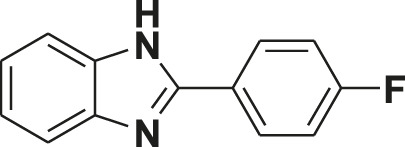	97.8 ± 0.7
2	MPD	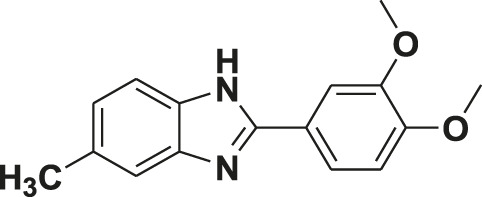	90.7 ± 3.3
3	AMPD1	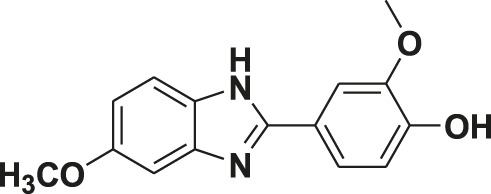	75.7 ± 4.9
4	TCPD	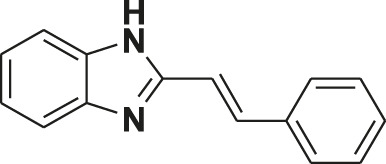	46.2 ± 5.1
5	CFPD	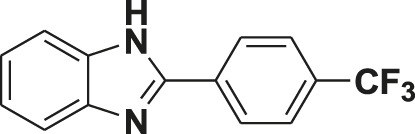	43.4 ± 5.7
6	VPD	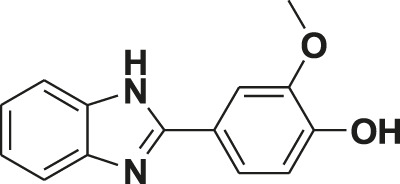	54.3 ± 6.1
7	23PD	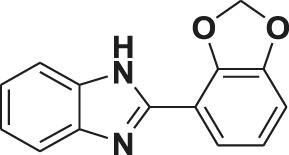	37.1 ± 4.6
8	MOPD	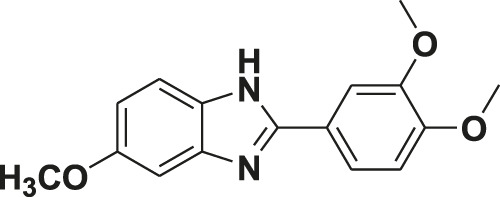	31.2 ± 6.2
9	Candesartan	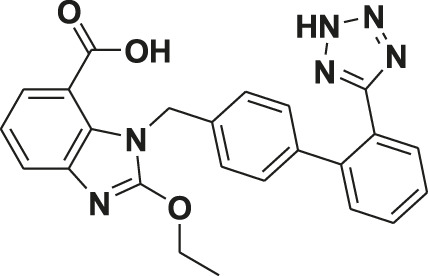	80.6 ± 6.5
10	Acetylcholine	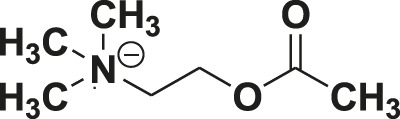	95.5 ± 0.7

### FPD Produced Potent Anti-hypertensive Activity in SHRs

**FIGURE 1:**
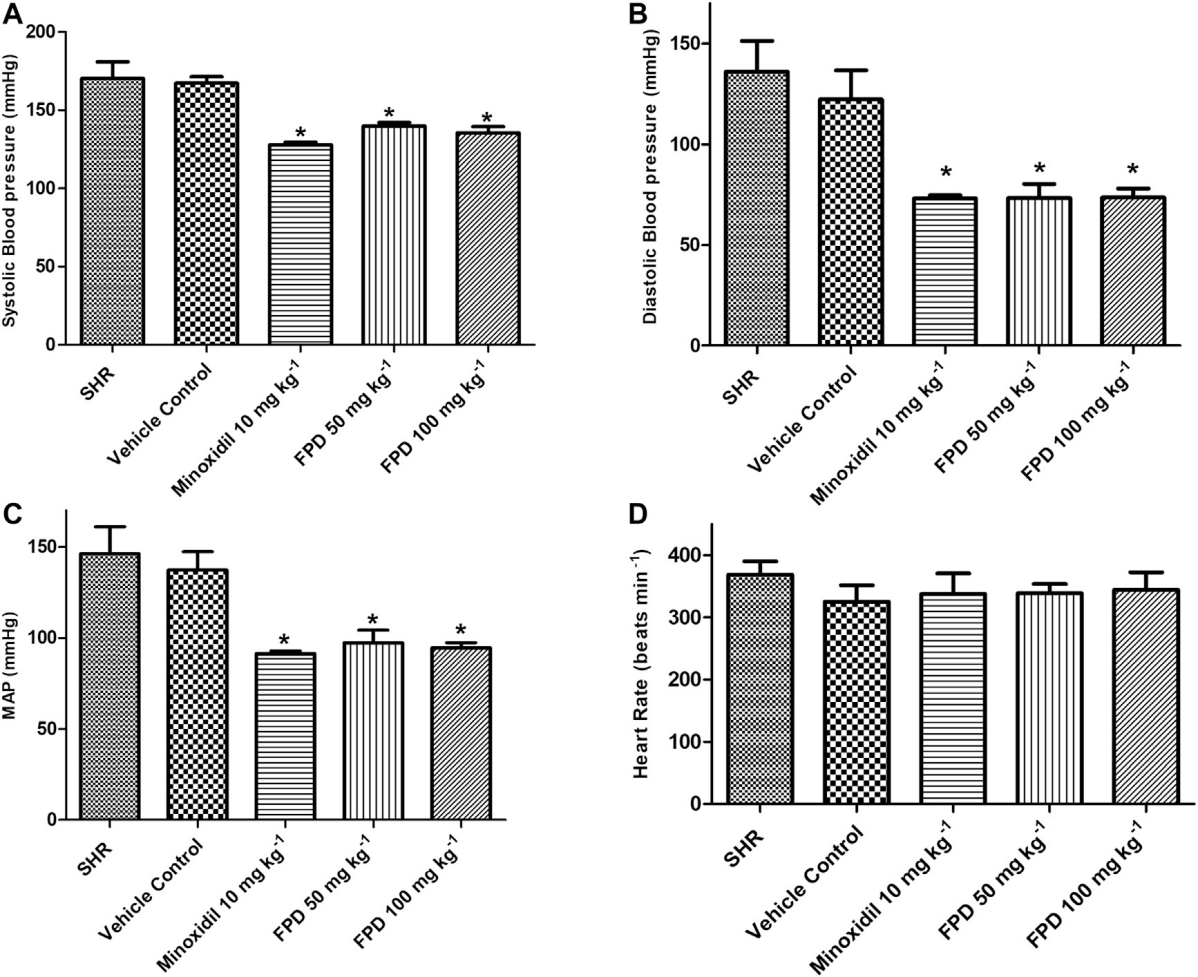
Effect of minoxidil (10 mg kg^−1^) and FPD (50 mg kg^−1^ and 100 mg kg^−1^) once daily for 14 days on SBP, DBP, MAP, and HR in spontaneously hypertensive rats **(A–D)** (mean ± S.E.M., *n* = 6; *p* < 0.05, * significance compared to SHR).

SHRs are one of the most commonly studied rodent models of hypertension for evaluating antihypertensive activity of pharmacophores in general and AT-1 receptor blockers from benzimidazoles like candesartan and telmesartan (Rizzoni et al., 1998; Cheng et al., 2018). In the present study, the novel fluorophenyl benzimidazole (FPD) was evaluated for *in vivo* antihypertensive activity in SHRs. Oral administration of FPD at 50 and 100 mg kg^−1^ for 14 days showed a significant decrease in SBP, DBP, and MAP as compared to vehicle control SHRs and are presented in Figure 1. Minoxidil (10 mg kg^−1^)-treated SHRs also showed a significant decline in hemodynamic parameters when compared with the control SHRs. The heart rate of treated rats remained unaffected compared to control (Figure 1D).

### Role of AT1 Receptor, cGMP Buildup, and Opening of BK_Ca_ Channel in FPD-Induced Vasorelaxation

**FIGURE 2:**
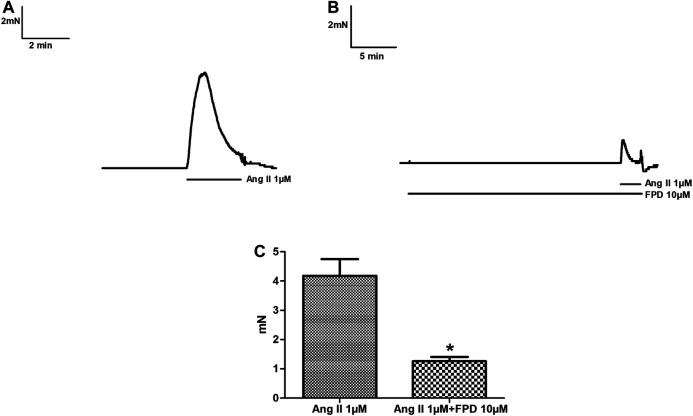
Traces showing angiotensin II (1 µM)-induced contraction in isolated rat superior mesenteric arterial rings **(A)** and in a pre-incubated arterial ring with FPD (10 µM) **(B)**. Contraction is expressed as the mean ± S.E.M. (*n* = 6); *p < 0.05 compared to control **(C)**.

Benzimidazoles as a class are known to produce antihypertensive activity through blockade of the AT1 receptor. In the present study, the AT1 receptor blocking potential of FPD was evaluated with angiotensin II-induced contraction pre-incubated with or without FPD and a significant blockade to angiotensin II-induced contraction was observed (Figure 2).

**FIGURE 3:**
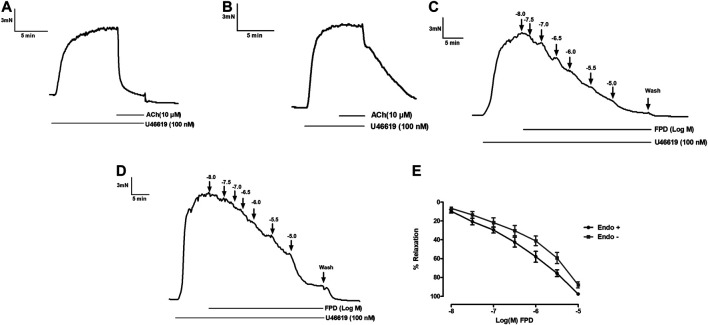
Traces showing acetylcholine (10 µM)-induced relaxation in endothelium-intact **(A)** and endothelium-denuded **(B)** isolated rat superior mesenteric arterial rings pre-constricted with U46619 (100 nM). Traces showing FPD-induced concentration dependent relaxation in endothelium-intact **(C)** and endothelium-denuded **(D)** arterial rings pre-constricted with U46619 (100 nM). E shows the sigmoidal concentration response curve obtained in **(C, D)**. Relaxation is expressed as the mean ± S.E.M. (*n* = 8) percentage reversal of U46619-induced contraction.

**FIGURE 4:**
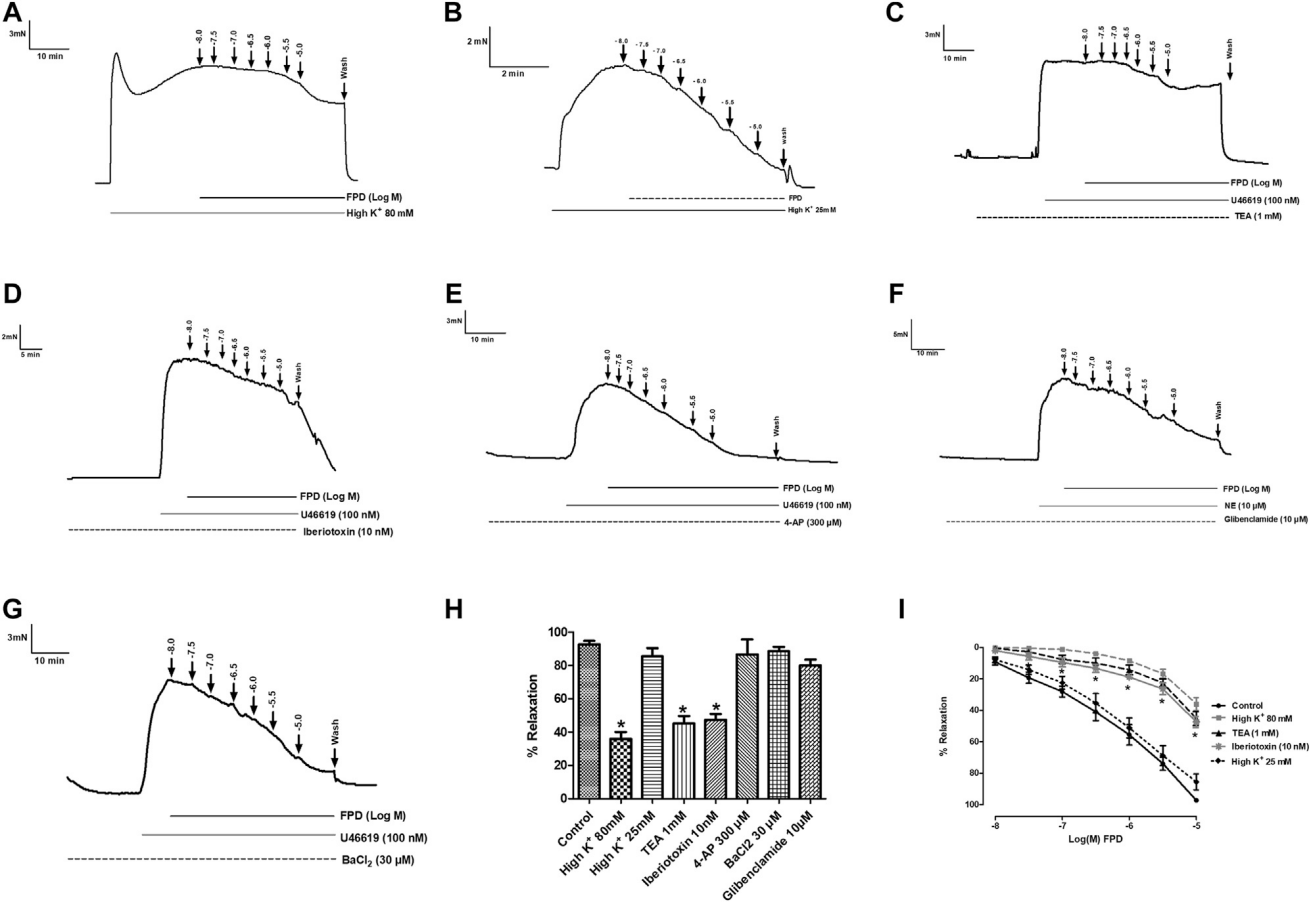
Traces showing FPD-induced concentration dependent relaxation in rat superior mesenteric arterial rings pre-constricted with 80 mM of high K^+^ MKHS **(A)** and 25 mM of high K^+^ MKHS **(B)**. Traces showing FPD-induced concentration dependent relaxation in arterial rings pre-incubated with potassium channel blockers; tetraethylammonium (TEA, 1 mM), iberiotoxin (10 nM), 4-aminopyridine (4-AP, 300 µM), glibenclamide (10 µM), and barium chloride (BaCl_2_, 30 µM) for 20 min and pre-constricted with U46619 (100 nM)/NE (10 µM) **(C–G)**. Relaxation is expressed as the mean ± S.E.M. (*n* = 6; *p < 0.05 compared to control and 25 mM of high K^+^ MKHS) percentage reversal of contraction **(H)**. **(I)** shows the sigmoidal concentration response curve obtained in **(A−D)**.

To study the role of the endothelium, the vasodilation response of FPD was evaluated using endothelium-intact and -denuded mesenteric arterial rings. Endothelium-denuded arterial rings produced no or less than 10% relaxation response to acetylcholine (10 µM) (Figures 3A,B). The concentration-dependent relaxation response to FPD in both endothelium-intact and -denuded rings showed no statistical difference in E_max_ (95.7 ± 1.2%; *n* = 12 for endothelium-intact and 88.9 ± 4.9%; *n* = 8 for endothelium-denuded mesenteric arterial rings) and pD2 (6.3 ± 0.1; *n* = 12 for endothelium-intact and 5.8 ± 0.1; *n* = 8 for endothelium-denuded mesenteric arterial rings) values (Figures 3C–E) suggesting endothelium-independent relaxation in the rat superior mesenteric artery. There are only a few reports on the modulation of potassium channel function by benzimidazoles (Lloyd et al., 2009) and no report is available on fluorobenzimidazole. Interestingly, when the superior mesenteric arterial rings were exposed to 80 mM of high K^+^ MKHS to prevent potassium channel-mediated hyperpolarization, the relaxation response to FPD was significantly diminished, but a contraction response with a lower concentration of K^+^ (25 mM of KCl containing MKHS) showed a complete concentration-dependent relaxation response to FPD suggesting an opening of potassium channels (Figures 4A,B). The involvement of sub-types of K^+^ channels in FPD-induced relaxation was studied in agonist (U46619/norepinephrine)-constricted arterial rings and is presented in Figures 4C–G. The results showed that pre-incubation of arterial rings with either TEA (1 mM) or iberiotoxin (10 nM) (Ca^2+^-activated K^+^ channel blocker; BK_Ca_ channel) abolished the relaxation response significantly in E_max_ (95.7 ± 1.2%; *n* = 12 for control to 45.3 ± 5.4%; *n* = 6 for 1 mM of TEA and 47.4 ± 3.5%; *n* = 6 for 10 nM of iberiotoxin treated mesenteric rings) and pD2 (6.3 ± 0.1; *n* = 12 for control to 4.8 ± 0.1; *n* = 6 for 1 mM of TEA and 4.8 ± 0.1; *n* = 6 for 10 nM of iberiotoxin treated mesenteric rings) values and are presented in Figures 4H,I.

**FIGURE 5:**
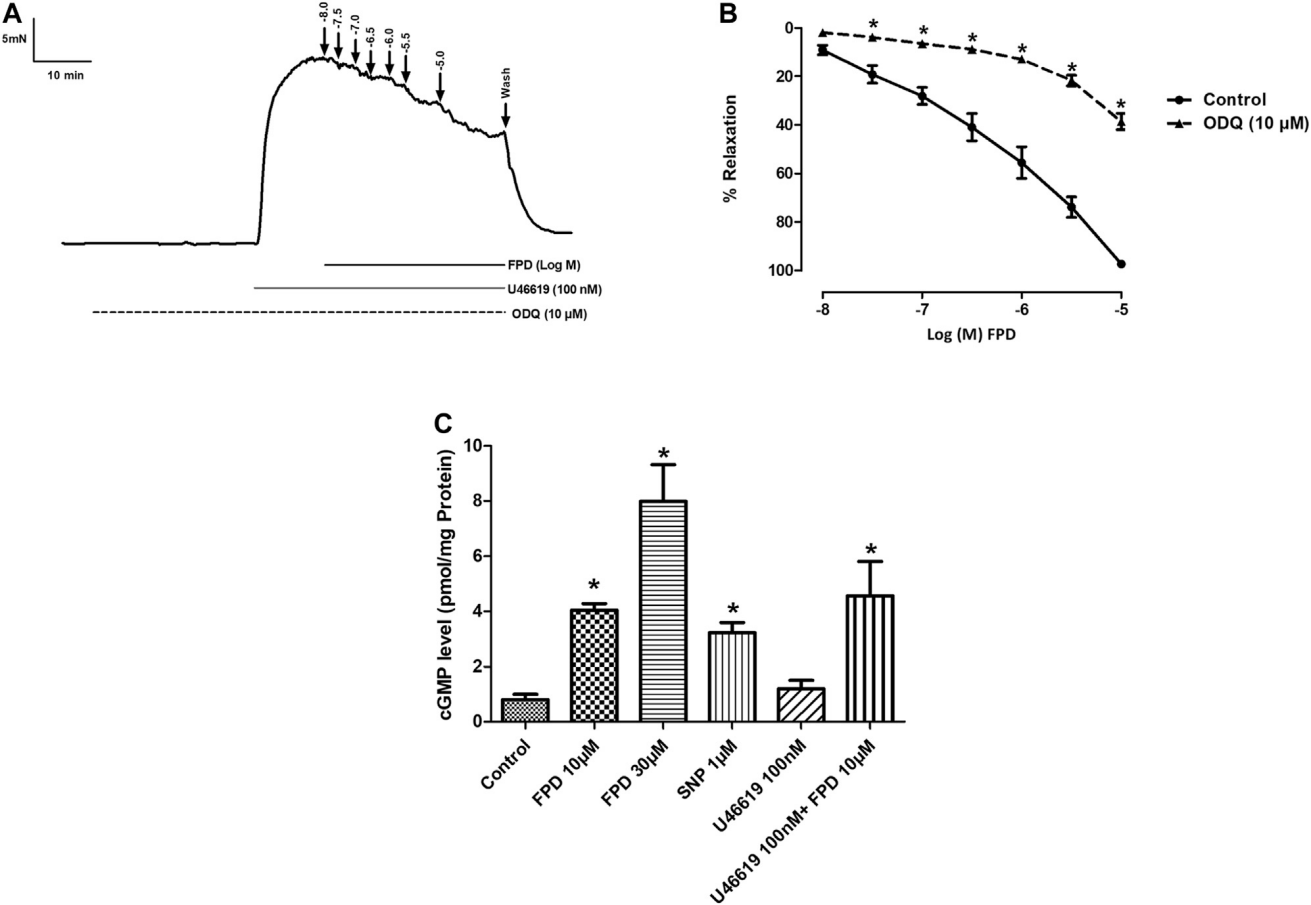
Traces showing FPD-induced concentration dependent relaxation in rat superior mesenteric arterial rings pre-incubated with ODQ (10 µM) for 20 min and pre-constricted with U46619 (100 nM) **(A)**. Concentration response curve of FPD in the presence of ODQ (10 µM) **(B)**. Relaxation is expressed as the mean ± S.E.M. (*n* = 6) percentage reversal of contraction. The bar diagram **(C)** depicts the mean intracellular cGMP level in treated rat superior mesenteric arterial tissues. Vertical bar represents mean ± S.E.M. (*n* = 6; *p < 0.05 compared to control).

The involvement of the sGC-cGMP-PKG pathway was also studied in the vasodilation response of FPD in the superior mesenteric artery. Pre-incubation of mesenteric arterial rings with ODQ for 20 min significantly abated the effect of FPD with a decrease in E_max_ (from 95.7 ± 1.2%; *n* = 12 for control to 38.6 ± 3.3%; *n* = 12 for 10 µM of ODQ treated mesenteric rings) and pD2 (6.3 ± 0.1; *n* = 12 for control and 4.6 ± 0.1; *n* = 12 for 10 µM of ODQ treated mesenteric rings) values (Figures 5A,B). Further, FPD treatment of the arterial tissues showed a concentration-dependent increase in tissue cGMP level 5.05 fold at 10 μM and 9.97 fold at 30 µM compared to control in the cGMP ELISA assay. SNP (1 µM) also increased the cGMP level 4.03 fold compared to control (Figure 5C).

#### FPD Enhanced Thallium Ion-Induced Fluorescence in Rat Aortic Smooth Muscle Cells

**FIGURE 6:**
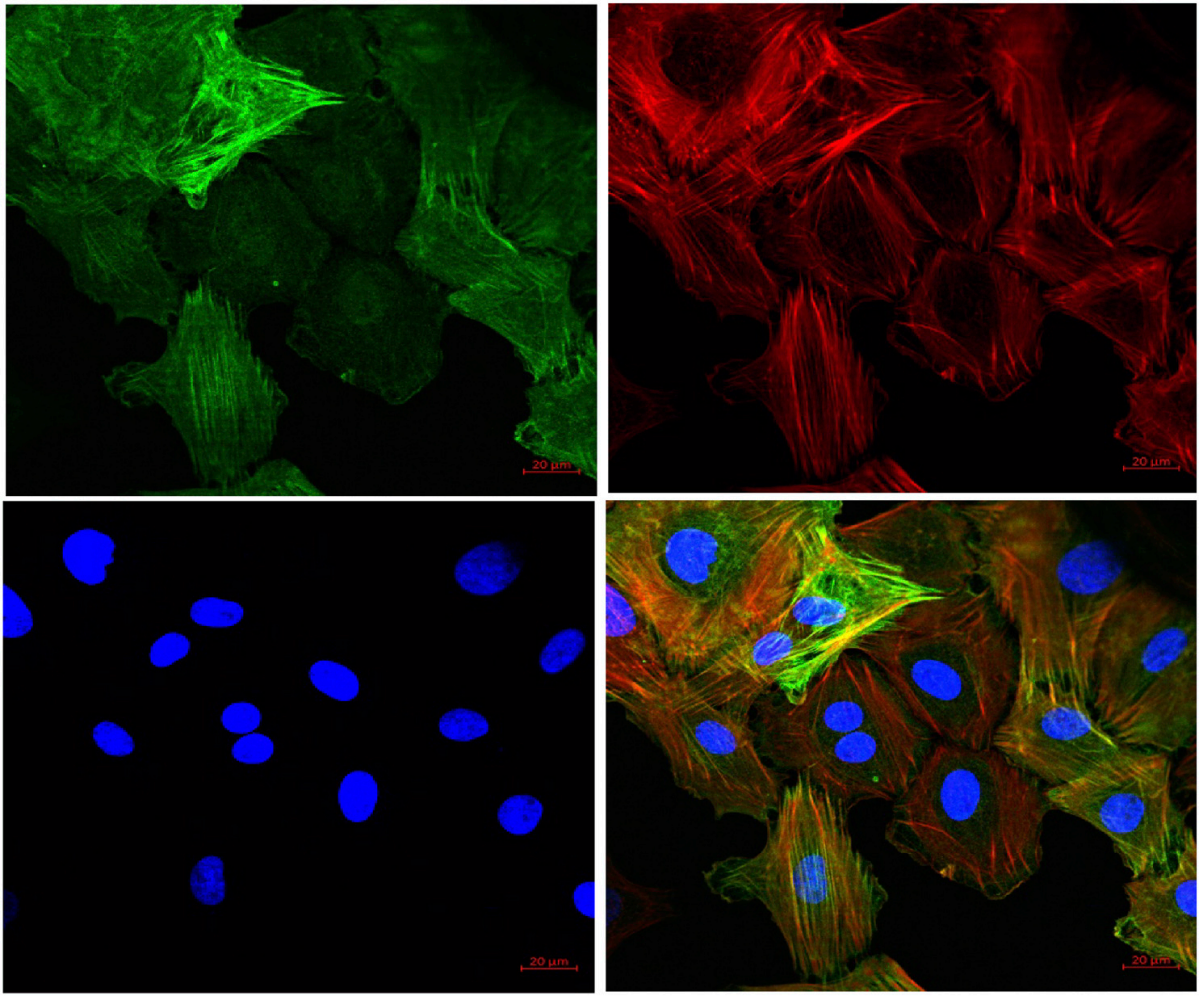
Representative pictures of cells derived from rat aorta stained with markers for VSMCs. Mesenchymal marker alpha-smooth muscle actin (α-SMA) (green) and F-actin (red) were stained positive in VSMCs. The nucleus of VSMC was stained by DAPI (blue) (40X).

**FIGURE 7:**
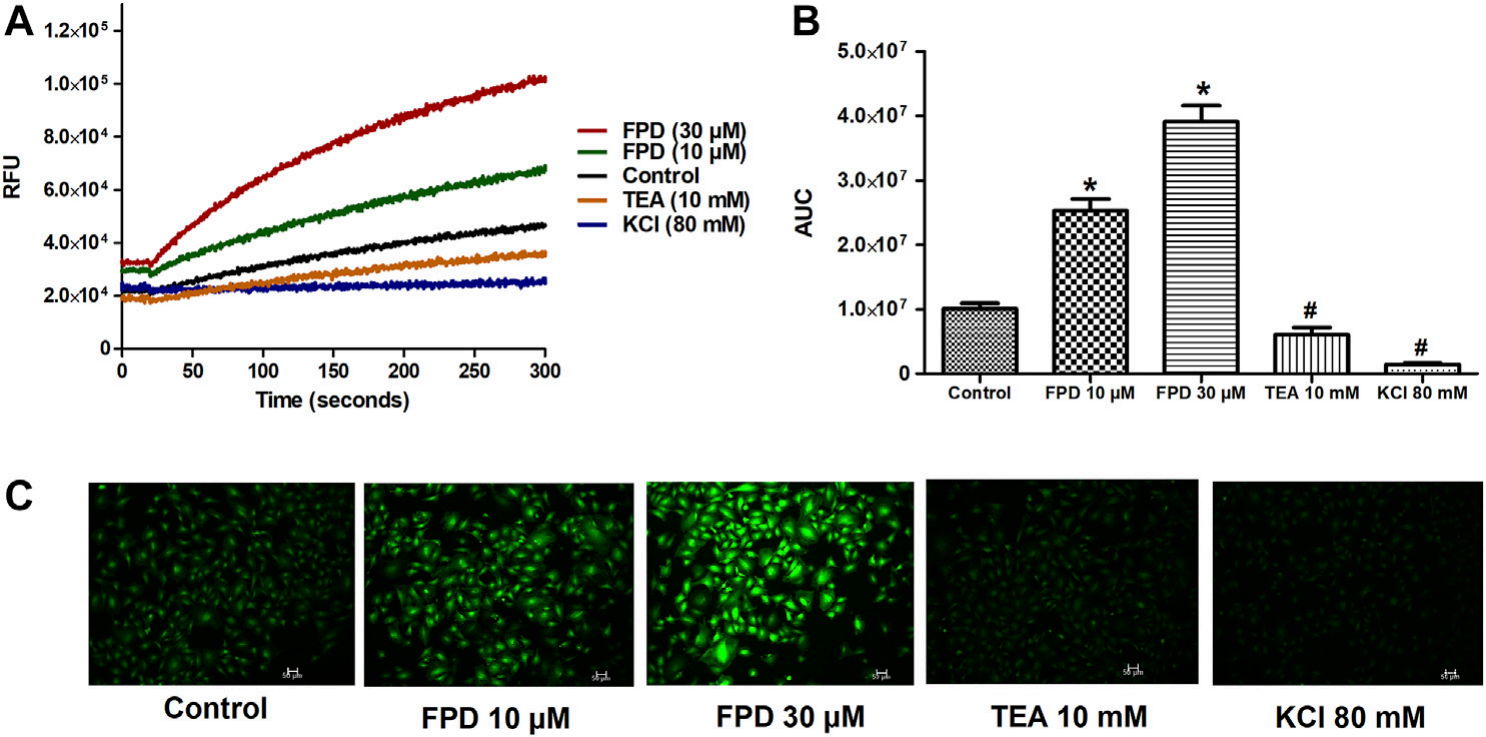
Representative traces of intracellular K^+^ concentration stimulated/inhibited by FPD (10 µM), FPD (30 µM), TEA (10 mM), and KCl (80 mM) in rat aortic VSMC loaded with FluxOR II Green **(A)**. **(B)** depicts the area under the curve (AUC; * significantly increased compared to control; # significantly decreased compared to control; *n* = 10). C shows the representative images of the cells.

To further substantiate our *ex vivo* observations, an *in vitro* potassium channel function assay was performed in rat aortic VSMC. Before conducting this assay, the characterization of VSMC was carried out using immunofluorescence staining and it was clearly seen that mesenchymal marker α-SMA and F-actin were stained positive (Figure 6). In the potassium channel function assay, it was seen that cells pretreated with FPD at 10 and 30 µM for 20 min showed a significant increase in relative fluorescence unit as compared to control, which clearly suggests that FPD led to the opening of the potassium channel, in which there is an inward movement of thallium ions which binds to the cytosolic residing fluorescent dye and results in increased fluorescence. Moreover, the exposure of cells to 10 mM of TEA and 80 mM of KCl was found to significantly decrease the fluorescence (Figures 7A–C).

#### FPD Blocks Bay K8644-Sensitive VDCC in Rat Mesenteric Arteries and Reduces Intracellular Ca^2+^ Level in Rat Aortic Smooth Muscle Cells

**FIGURE 8:**
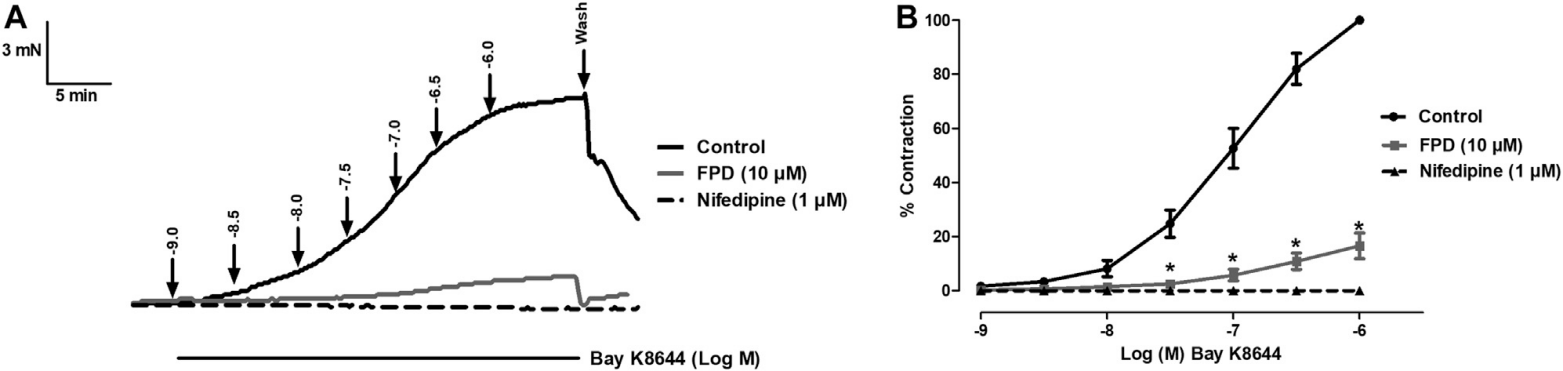
Tracings of concentration dependent Bay K8644-induced contraction alone and in the presence of FPD (10 µM) and nifedipine (1 µM) in isolated rat superior mesenteric artery **(A)**. Concentration response curve of Bay K8644 alone, in combination with FPD (10 µM for 20 min) and nifedipine (1 µM) **(B)** (mean ± S.E.M., *n* = 8; *p < 0.05 compared to control).

**FIGURE 9:**
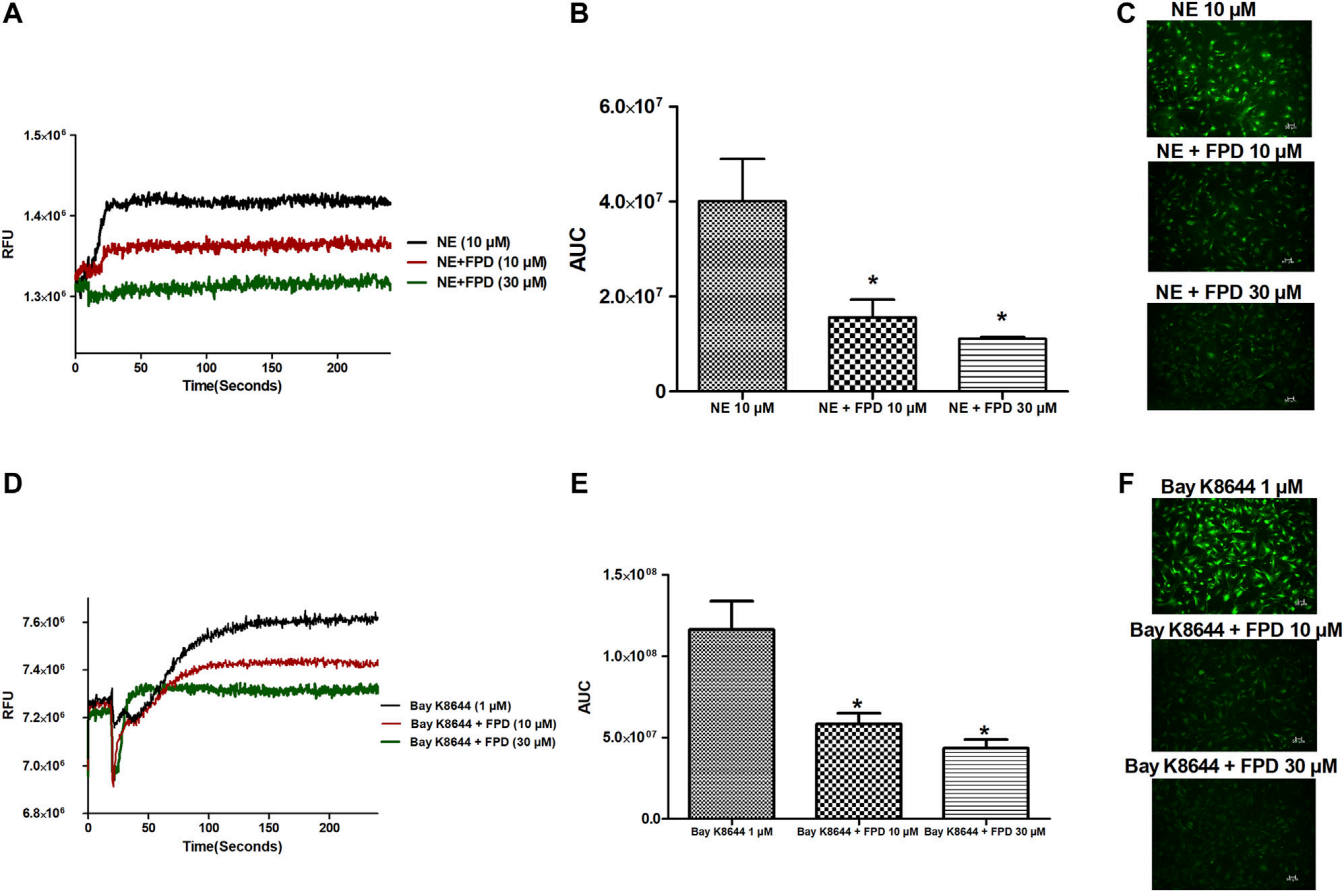
Representative traces of cytosolic Ca^2+^ concentration ([Ca^2+^]_i_) stimulated by NE (10 µM), Bay K8644 (1 µM) alone, and in the presence of FPD (10 and 30 µM) in rat aortic VSMC loaded with Fluo-4 **(A, D)**; area under the curve **(B, E)** (AUC, * significantly decreased compared to NE/Bay K8644 treated cells; *n* = 10); representative images of the cells **(C, F)**.

As the relaxation response of FPD was not completely abolished either in the presence of high potassium depolarizing MKHS (∼60%), TEA, or ODQ, the contribution of other targets like VDCC was explored to study the mechanism of vasorelaxation in the mesenteric artery. We used a specific L-type VDCC opener, Bay K8644, to study the involvement of VDCC in the vasodilation response of FPD in the superior mesenteric artery. Cumulative concentration-dependent contraction response of Bay K8644 from 1 nM to 1 µM was elicited in slightly depolarized (K^+^, 15 mM) arterial rings (Figure 8A). Pre-incubation of arterial rings with FPD (10 µM) or a known blocker of VDCC, nifedipine (1 µM) independently for 20 min significantly abolished the contraction response induced by Bay K8644 (E_max_ 100 ± 0.0%; *n* = 8 control, 16.6 ± 4.7%; *n* = 8 for FPD (10 µM) and 0.0 ± 0.0%, *n* = 5 for nifedipine (1 µM) treated mesenteric rings) and pD2 (7.1 ± 0.0; *n* = 8 for control and 4.7 ± 0.4; *n* = 8 for FPD (10 µM) treated mesenteric rings) values (Figures 8A,B). To investigate the effect of FPD on intracellular Ca^2+^ level by inhibiting calcium channel function, a calcium fluorescence assay was carried out in the VSMC using Fluo-4 AM dye which strongly fluoresces when there is a rise in intracellular calcium concentration. In this experiment, we used NE (10 µM) to induce VSMC [Ca^2+^]_i_ and it was found that calcium elevation by NE (10 µM) was significantly decreased in the FPD-treated cells at the concentration of 10 and 30 μM, this is illustrated in Figures 9A–C. To further substantiate our finding that FPD blocks L-type VDCC; the cells were treated with Bay K8644 (1 µM) and it was found that a rise of intracellular calcium concentration by Bay K8644 was significantly reduced in the FPD (10 and 30 µM) pretreated cells (Figures 9D–F).

### *In silico* Docking of FPD With AT1, BK_Ca_ Channels, VDCC, and sGC

**FIGURE 10:**
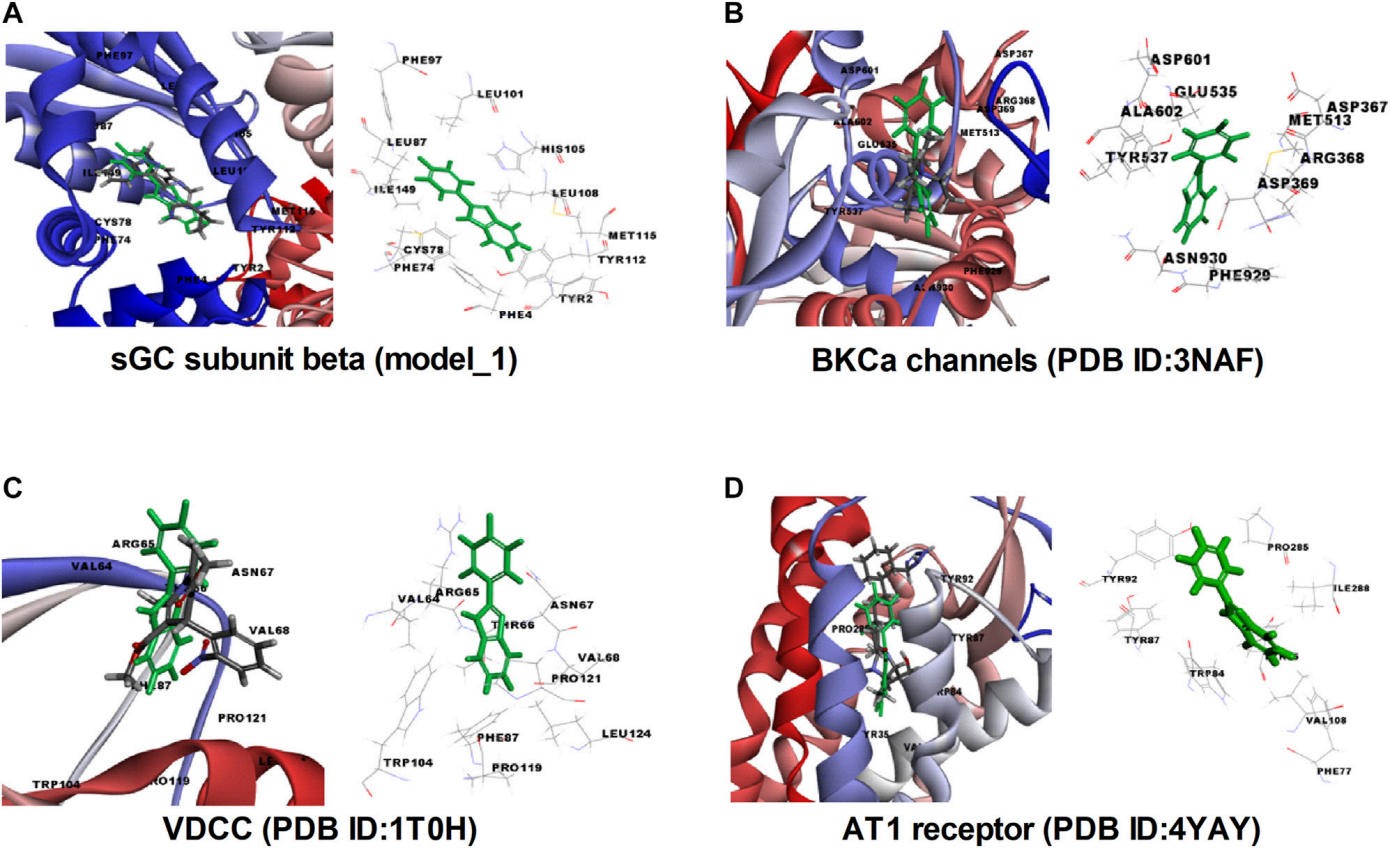
Molecular insight, interactive amino acid residues, and binding site conformation of FPD (green) with control compound (element color) docked on sGC subunit β (homology model_1). **(A)**, BK_Ca_ channels (PDB ID: 3NAF). **(B)** VDCC (PDB ID: 1T0H). **(C)** angiotensin II receptor 1 (PDB ID: 4YAY). **(D)**.

In order to validate our *ex vivo*, *in vitro*, and *in vivo* observation of potent vasorelaxation, cGMP buildup in arterial tissues, potassium and calcium channel function modulation, and antihypertensive activity with FPD, we docked our compound with selected target proteins, i.e., BK_Ca_ channels (PDB ID: 3NAF), VDCC (PDB ID: 1T0H), angiotensin II receptor 1 (PDB ID: 4YAY), and with a freshly modeled β subunit of sGC (model_1; Supplementary Figure S3). Docking of FPD with the selected targets resulted in a LibDock score of 63.87, 63.56, 60.55, and 69.15 for AT1 receptor, BK_Ca_ channel, VDCC, and sGC β, respectively. The docking scores suggested that the compound had a fair chance of binding with all the selected protein targets. The results of the docking studies are summarized in Figure 10 and Table 2. The analysis of the protein-ligand complexes revealed the interactive amino acid residues and binding site conformation of compound FPD docked on the specified targets.

**TABLE 2:** LibDock scoring functions, hydrogen bonding, Pi-interaction, and active binding site residues observed in the in silico docking studies of FPD with selected targets: sGC subunit beta (homology model_1), BK_Ca_ channels (PDB ID:3NAF), VDCC (PDB ID:1T0H), and angiotensin II receptor 1 (PDB ID:4YAY) using reported target specific ligands as a standard compound.

Target	PDB ID/homology model	Ligands FPD/control	LibDock score	-H Bonding	Pi-interaction	Amino acid residues
sGC subunit beta	Model_1	FPD	69.15	No	PHE4 PHE74	TYR2, PHE4, PHE74, CYS78, LEU87, PHE97, LEU101, HIS105, LEU108, TYR112, MET115, ILE149
		CID:9798973 (Bay 41–2,272)	123.36	No	No	TYR2, PHE4, VAL5, PHE74, PHE75, CYS78, LEU87, LEU101, HIS105, LEU108, TYR112, MET115, PRO118, PHE120, ILE145, VAL146, ILE149
BKca channels	3NAF	FPD	63.56	No	ARG368	ASP367, ARG368, ASP369, MET513, GLU535, TYR537, ASP601, ALA602, PHE929, ASN930
		CID:5,946 (Tetraethy-lammonium chloride)	50.71	No	No	ARG368, ASP369, MET513, GLU535, TYR537, ALA602, PHE929, ASN930
VDCC	1T0H	FPD	60.55	No	No	VAL64, ARG65, THR66, ASN67, VAL68, PHE87, TRP104, PRO119, PRO121, LEU124
		CID:4,485 (nifedipine	78.56	No	No	VAL64, ARG65, THR66, ASN67, VAL68, LEU93, TRP104, ILE118, PRO119, PRO121, LEU124
Angiotensin II receptor 1	4YAY	FPD	63.87	No	TYR92	TYR35, PHE77, TRP84, TYR87, TYR92, VAL108, PRO285, ILE288
		CID:2,541 (candesartan)	132.79	ARG167	No	ALA21, GLY22, ARG23, ILE31, TYR35, PHE77, TRP84, TYR87, THR88, TYR92, SER105, VAL108, ARG167, ILE172, VAL179, ASP281, MET284, PRO285, ILE288

### Acute and Sub-acute Oral Toxicity of FPD in Swiss Albino Mice

**FIGURE 11:**
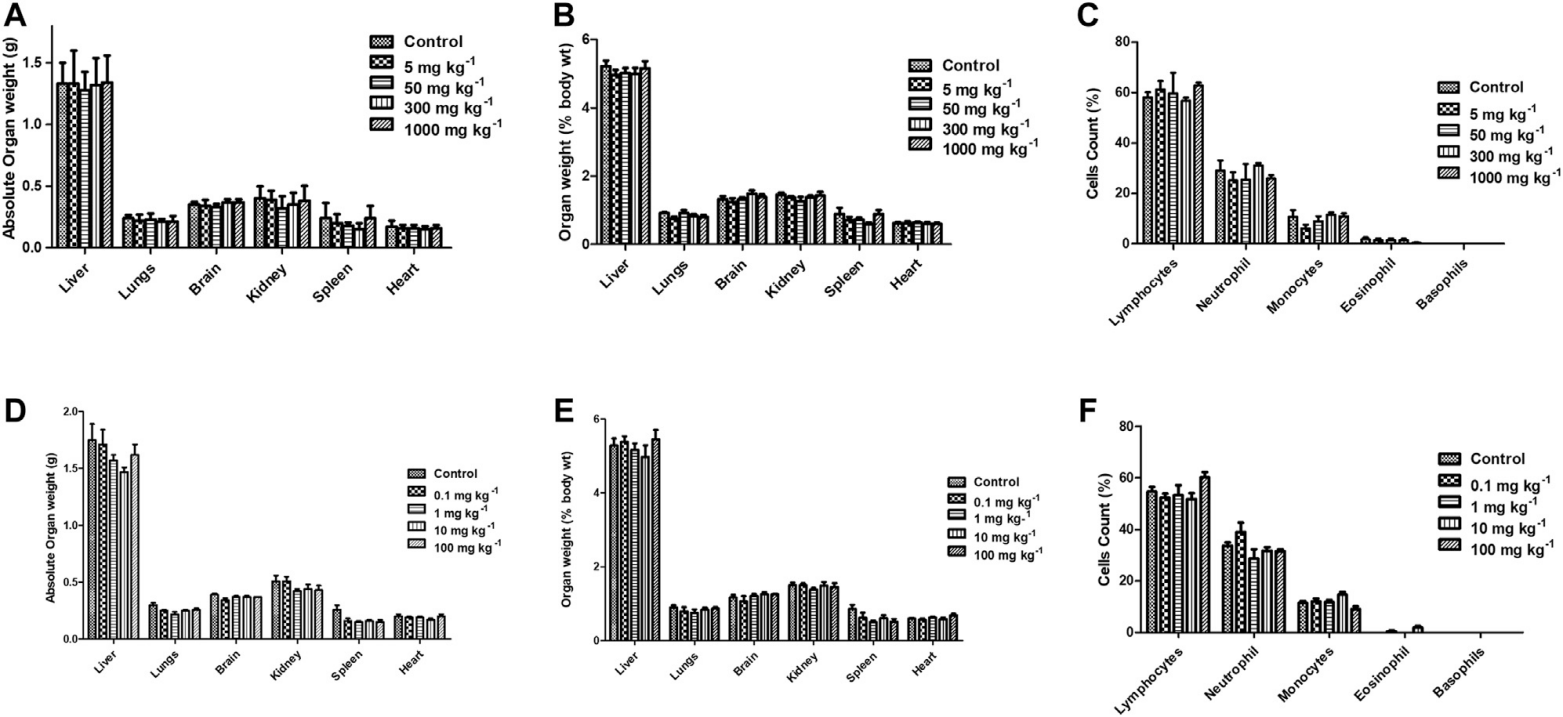
Effect of FPD as a single acute oral dose at 5, 50, 300, and 1,000 mg kg^−1^ on absolute organ weight, relative organ weight, and differential leukocytes count (DLC) **(A–C)** in Swiss albino mice (mean ± SEM, *n* = 6). Effect of FPD as sub-acute oral dose at 0.1, 1, 10, and 100 mg kg^−1^ once orally for 28 days on absolute organ weight, relative organ weight, and DLC **(D–F)** in Swiss albino mice (mean ± S.E.M., *n* = 6).

Acute and sub-acute oral toxicity studies with FPD in Swiss albino mice showed no mortality, morbidity, and no change in gait or posture throughout the experimental period. Blood and serum samples upon analysis did not show any significant changes in the hematological and biochemical parameters studied, and are presented in Table 3. Animals on gross pathological examination showed non-significant changes in physical appearances, texture, and absolute and relative weights of vital organs, the details are shown in Figure 11. These observations suggested that FPD was well tolerated by Swiss albino mice up to 1,000 mg kg^−1^ in body weight as a single acute oral dose and up to 100 mg kg^−1^ as a repeated oral dose for 28 days.

**TABLE 3:** Effect of FPD as a single acute oral dose at 5, 50, 300, and 1,000 mg kg^−1^ and as a sub-acute oral dose at 0.1, 1, 10, and 100 mg kg^−1^ (once orally for 28 days) on body weight and hematological and serum biochemical parameters in Swiss albino mice (mean ± SEM; *n* = 6).

Parameters	Dose of FPD at mg kg^−1^ body weight as a single oral dose					Dose of FPD at mg kg^−1^ body weight as a repeated oral dose				
	Control	5 mg kg^−1^	50 mg kg^−1^	300 mg kg^−1^	1,000 mg kg^−1^	Control	0.1 mg kg^−1^	1 mg kg^−1^	10 mg kg^−1^	100 mg kg^−1^
Body weight (gm)	26.92 ± 1.79	28.00 ± 1.57	25.53 ± 0.95	25.28 ± 2.13	26.57 ± 1.21	33.70 ± 2.14	32.20 ± 2.95	30.12 ± 0.96	29.45 ± 1.15	29.48 ± 0.60
Hemoglobin (gm dL^−1^)	13.11 ± 0.97	14.02 ± 0.57	15.31 ± 0.49	14.77 ± 0.65	14.33 ± 0.47	16.64 ± 0.65	17.86 ± 0.76	17.20 ± 0.33	17.68 ± 0.68	16.88 ± 0.38
RBC (million mm^−3^)	6.62 ± 1.02	6.60 ± 0.72	6.61 ± 0.27	6.67 ± 0.81	6.01 ± 0.11	9.30 ± 1.27	8.97 ± 1.19	9.93 ± 0.35	9.80 ± 0.29	9.86 ± 0.23
WBC (thousands mm^−3^)	4.56 ± 0.27	5.30 ± 0.55	4.33 ± 0.13	4.85 ± 0.31	5.10 ± 0.55	4.30 ± 0.23	4.71 ± 0.26	3.78 ± 0.46	4.12 ± 0.44	4.41 ± 0.33
ALP (U L^−1^)	343.04 ± 25.44	340.11 ± 62.96	332.99 ± 73.78	340.66 ± 70.62	331.71 ± 34.01	203.34 ± 5.57	202.06 ± 9.42	208.66 ± 25.58	209.00 ± 8.52	200.82 ± 14.78
SGOT (U L^−1^)	22.08 ± 2.41	23.07 ± 2.27	21.35 ± 2.93	27.01 ± 3.56	26.97 ± 3.48	47.90 ± 10.12	48.82 ± 3.40	42.20 ± 4.54	42.65 ± 6.22	45.65 ± 7.40
SGPT (U L^−1^)	22.29 ± 2.14	25.28 ± 2.38	21.35 ± 2.82	20.14 ± 1.16	26.96 ± 3.67	22.93 ± 1.82	22.77 ± 4.30	18.26 ± 0.76	18.82 ± 0.58	21.08 ± 5.11
Creatinine (mg dl^−1^)	0.28 ± 0.01	0.35 ± 0.02	0.30 ± 0.03	0.35 ± 0.02	0.33 ± 0.03	0.32 ± 0.02	0.37 ± 0.01	0.30 ± 0.01	0.32 ± 0.01	0.31 ± 0.01
Triglycerides (mg dl^−1^)	122.87 ± 7.87	135.61 ± 3.74	127.28 ± 4.01	120.09 ± 3.10	124.00 ± 5.40	81.81 ± 4.75	77.54 ± 9.32	78.14 ± 7.35	78.23 ± 13.96	71.37 ± 3.42
Cholesterol (mg dl^−1^)	141.37 ± 4.75	136.06 ± 8.98	133.31 ± 4.46	144.97 ± 7.06	132.33 ± 2.44	165.38 ± 17.16	162.17 ± 16.19	169.24 ± 8.65	169.78 ± 6.10	162.95 ± 4.95
Bilirubin (mg dL^−1^)	0.37 ± 0.02	0.39 ± 0.02	0.33 ± 0.02	0.39 ± 0.03	0.41 ± 0.04	0.35 ± 0.01	0.39 ± 0.03	0.34 ± 0.01	0.42 ± 0.03	0.33 ± 0.03
Albumin (g dl^−1^)	2.06 ± 0.15	2.38 ± 0.14	2.11 ± 0.09	2.42 ± 0.12	2.02 ± 0.05	2.76 ± 0.11	2.88 ± 0.06	2.88 ± 0.11	3.22 ± 0.03	2.87 ± 0.02
Protein (mg ml^−1^)	5.93 ± 0.42	6.44 ± 0.25	6.77 ± 0.25	6.96 ± 0.24	6.70 ± 0.60	6.06 ± 0.27	6.28 ± 0.19	5.78 ± 0.16	6.39 ± 0.28	5.59 ± 0.05

## Discussion

The results of the present study demonstrate that 1) FPD produced an antihypertensive response in SHR animals; 2) FPD markedly inhibited Ang II-induced contraction in the rat superior mesenteric artery; 3) the relaxation response to FPD was independent of the endothelium; 4) nevertheless, the test compound increased tissue cGMP levels and the relaxation response elicited with FPD was significantly attenuated by sGC inhibitor ODQ; 5) FPD markedly inhibited L-type calcium channel agonist Bay K8644-induced contractions in the mesenteric arterial rings, as well as attenuated Bay K8644-stimulated Ca^2+^ influx in aortic smooth muscle cells; 6) BK_Ca_ channel blockers like iberiotoxin (10 nM) and TEA (1 mM) significantly inhibited FPD-induced relaxation in the mesenteric artery; 7) potassium channel opening by FPD was evident from florescence studies with VSMCs which showed FPD-induced increase in thallium ion entry and its blockade by TEA (10 mM) and KCl (80 mM); and 8) the test drug was well tolerated by Swiss albino mice up to 1,000 mg kg^−1^ in acute oral toxicity given as a single dose and up to 100 mg kg^−1^ in sub-acute oral toxicity when given once daily for 28 days.

Optimization of the substituents around the benzimidazole nucleus has resulted in many drugs including important antihypertensive molecules like candesartan, telmisartan, and cilexitil (Supplementary Figure S1A). Many benzimidazole-based compounds act as antihypertensives by intercepting the renin angiotensin aldosterone system (RAS) in which angiotensin II (Ang II), by acting on the AT1 receptor, causes vasoconstriction, Na^+^ retention, aldosterone release, and the development of hypertension. The aim of our designed pharmacophore was to obtain a small molecule antihypertensive agent on a benzimidazole core. The lead compound FPD (a C-substituted fluorophenyl benzimidazole) during detailed *ex vivo* studies in healthy Wistar rats showed AT1 receptor blocking activity and modulation of VDCC function, which are greatly affected in *in vivo* models like SHRs, and in line with our hypothesis, the molecule exhibited strong antihypertensive activity in SHRs. The molecule in the present study is much smaller C-substituted fluorophenyl benzimidazole and currently no such report is available on its antihypertensive activity. Recently the synthesis, characterization, and antihypertensive activities of 2-phenyl-substituted benzimidazoles and aminocarbonyl benzimidazoles were reported (Khan et al., 2018; Wu et al., 2019). The AT1 receptor blocking action of FPD in the rat superior mesenteric artery, as demonstrated in the present investigation, appears to be consistent with its antihypertensive action in an SHR model of hypertension. In agreement with our observations, the antihypertensive activity and AT1 receptor blocking activity of benzimidazoles like candesartan (Nagai et al., 2004) and substituted benzimidazoles like 5-nitro benzimidazoles (Shah et al., 2008), and 1, 4 or 1, 5 di-substituted benzimidazoles (Zhu et al., 2014) were also reported. SHR are known to have a very high level of angiotensin II expression and downstream AT1 receptor activation for the induction of high blood pressure (Mao and Li, 2015) and hence AT1 receptor blockers were found to be the most frequently reported antihypertensive agent in these models (Choisy et al., 2015).

Regarding the contribution of the vascular endothelium, the observations of the present study show that FDP-induced relaxation of the superior mesenteric artery was endothelium-independent. Our finding is consistent with an earlier report, which demonstrated endothelium-independent vasodilation response of 2-substituted 1H benzimidazoles in rat aorta (Estrada-Soto et al., 2006).

Intracellular rise in cGMP level in vascular smooth muscle and downstream activation of G kinase are well established mechanisms for vasodilation response, and antihypertensive activity of a number of pharmacophores, including NO and NO donors. Blocking of vasodilation response of FPD by ODQ indicates the involvement of sGC in the smooth muscle relaxation response. This is further supported by the tissue cGMP buildup in mesenteric arteries upon treatment with FPD. Antihypertensive benzimidazoles like candesartan were found to enhance cGMP buildup in heart tissue from an ischemia and reperfusion-induced model of cardiac infarction in mongrel dogs (Jugdutt et al., 2000).

The sensitivity of relaxation response of FPD in superior mesenteric arteries to high K^+^, TEA, and iberiotoxin suggested the involvement of the BK_Ca_ channel in the vasodilation response, which was further confirmed by the observation that TEA and KCl (80 mM) blocked thallium ion entry into aortic smooth muscle cells. Additional evidence for the involvement of BK_Ca_ channels in the vasorelaxation mechanism of FPD comes from the *in silico* studies showing a decent docking score. However, an electrophysiological study with FPD using a patch clamp would provide a more direct evidence of the modulation of BK channel function by FPD. The rise of intracellular cGMP level in vascular smooth muscle and its close association in the functional modulations of the BK_Ca_ channel has been reported by several workers including us (Chanda et al., 2016; Liu et al., 2016).

The present study also identified VDCC as a putative target for FPD contributing to vasorelaxation response in superior mesenteric arteries and also in antihypertensive activity in SHRs. FPD significantly blocked Bay K8644-induced contractile response in a mesenteric artery, and also Bay K8644-stimulated Fluo-4-fluorescence in rat VSMC. Docking studies of FPD with the L-type calcium channel are also in strong agreement with the *ex vivo* and *in vitro* observations. Although literature is available for benzimidazole molecules like NS-649 (Varming et al., 1996) and mibefradil for calcium channel blocking activities, no such reports are available for fluorophenyl benzimidazoles. Mibefradil, a benzimidazolyl-substituted tetraline analogue, selectively blocks T-type voltage-gated plasma membrane calcium channels in vascular smooth muscle (Ernst and Kelly, 1998; Ball et al., 2009). However, like the BK_Ca_ channel, an electrophysiological study with FPD using a patch clamp would provide direct evidence of the blockade of VDCC by FPD.

In both the acute (FPD 100 mg kg^−1^ as single acute oral dose) and sub-acute oral toxicity studies in Swiss albino mice (FPD 100 mg kg^−1^ once daily for 28 days) the test drug did not exhibit any toxicity.

## Limitations

There are certain limitations in the present study. For example, we studied the antihypertensive action of FPD in SHRs, but the mechanistic work with respect to vasorelaxant mechanisms were studied in the superior mesenteric artery/aortic smooth muscle cells from Wistar rats. Further, future investigations using patch clamp studies are required to provide direct evidence for the involvement of L-type calcium and BK_Ca_ channels in the vasorelaxation mechanisms of FPD. The aim of the docking study was to determine whether FPD binds with selected targets with a decent docking score as we speculated that FPD binds the selected targets to produce biological activities in *ex vivo*, *in vitro,* and *in vivo* models. However, detailed *in silico* docking of FPD to the selected target proteins at their biologically active pockets may be studied to understand the specific effect of FPD in its targets which were not explored in the present study.

## Conclusion

The present study demonstrates that FPD (C-fluorophenyl benzimidazole), a novel AT1 receptor blocker possessed antihypertensive activity in the SHRs hypertension model. It is also suggested that an FPD-induced rise in tissue cGMP, blockade of L-type calcium channels, and opening of BK_Ca_ channels may significantly contribute to the vasorelaxation mechanisms.

## Data Availability

The raw data supporting the conclusions of this article will be made available by the authors, without undue reservation.

## Abbreviations

4-AP, 4-aminopyridine; DAPI, 4′,6-diamidino-2-phenylindole; DMSO, dimethylsulfoxide; FPD, fluorophenyl benzimidazole; High K+ MKHS, high potassium depolarizing solution; L-NAME, Nω-nitro-L-arginine methyl ester hydrochloride; MKHS, modified Krebs-Henseleit solution; ODQ, 1H-[1,2,4]oxadiazolo [4,3-a]quinoxalin-1-one; TCA, trichloroacetic acid; TEA, tetraethyl ammonium; U46619, thromboxane analogue.
