# Supplementary material for: Antihypertensive Effect of a Novel Angiotensin II Receptor Blocker Fluorophenyl Benzimidazole: Contribution of cGMP, Voltage-dependent Calcium Channels, and BKCa Channels to Vasorelaxant Mechanisms
Source: Front Pharmacol. 2021 Mar 30;12:611109. doi: 10.3389/fphar.2021.611109 (PMC8042648; doi:10.3389/fphar.2021.611109)
Supplement: Supplementary file 1 [file datasheet1.docx]

Figure S1: Clinically used drugs with benzimidazole core and novel benzimidazole, A. Schematic presentation for the preparation of various benzamidazoles, B.

**
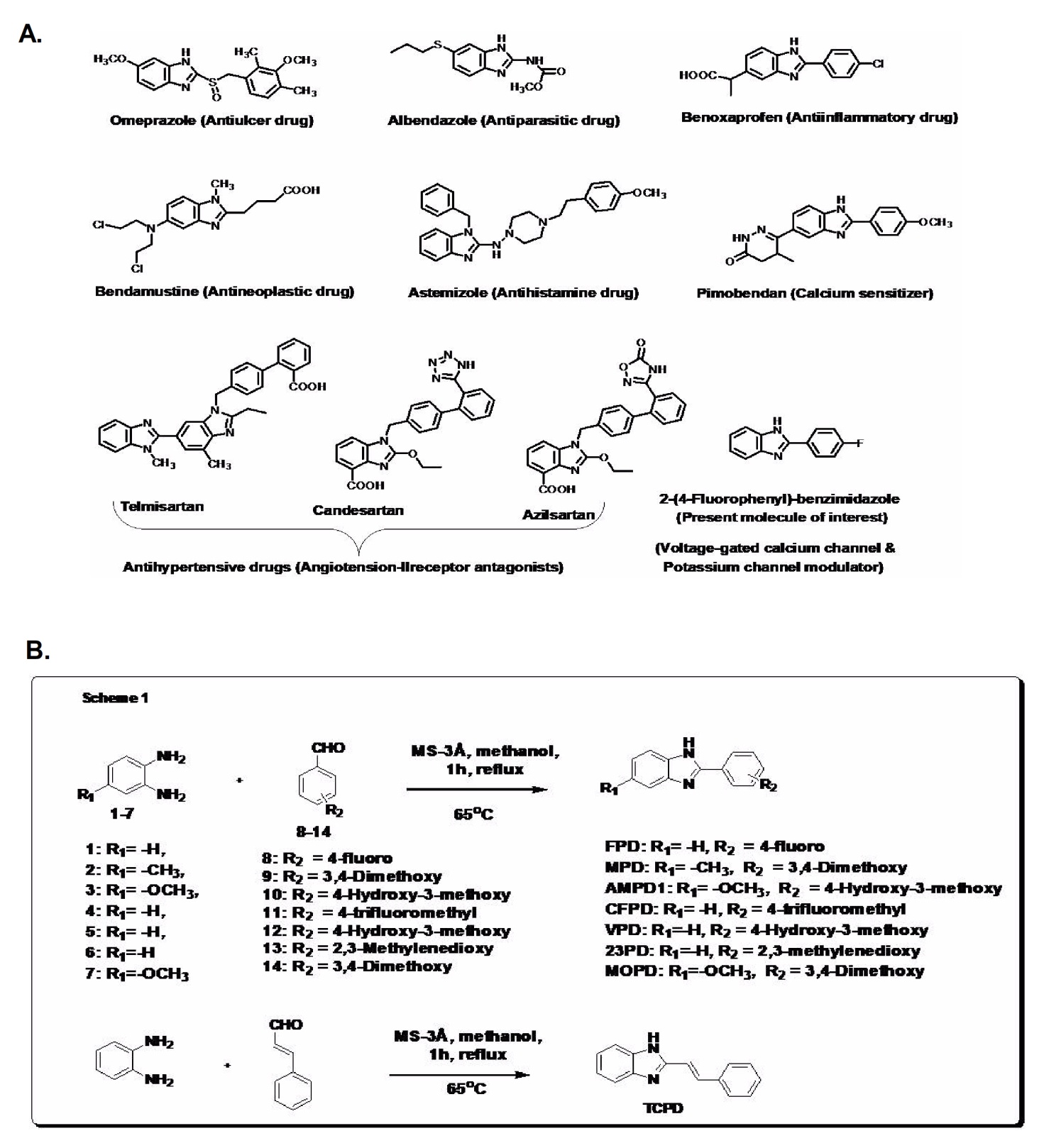
**

Figure S2: Traces showing FPD A, MPD B, AMPD1 C, -induced concentration dependent relaxation in endothelium intact isolated rat superior mesenteric arterial rings preconstricted with U46619 (100 nM). D shows the sigmoidal concentration response curve obtained in A-C. Relaxation is expressed as the Mean ± SEM, (n=3) percentage reversal of U46619-induced contraction.

**
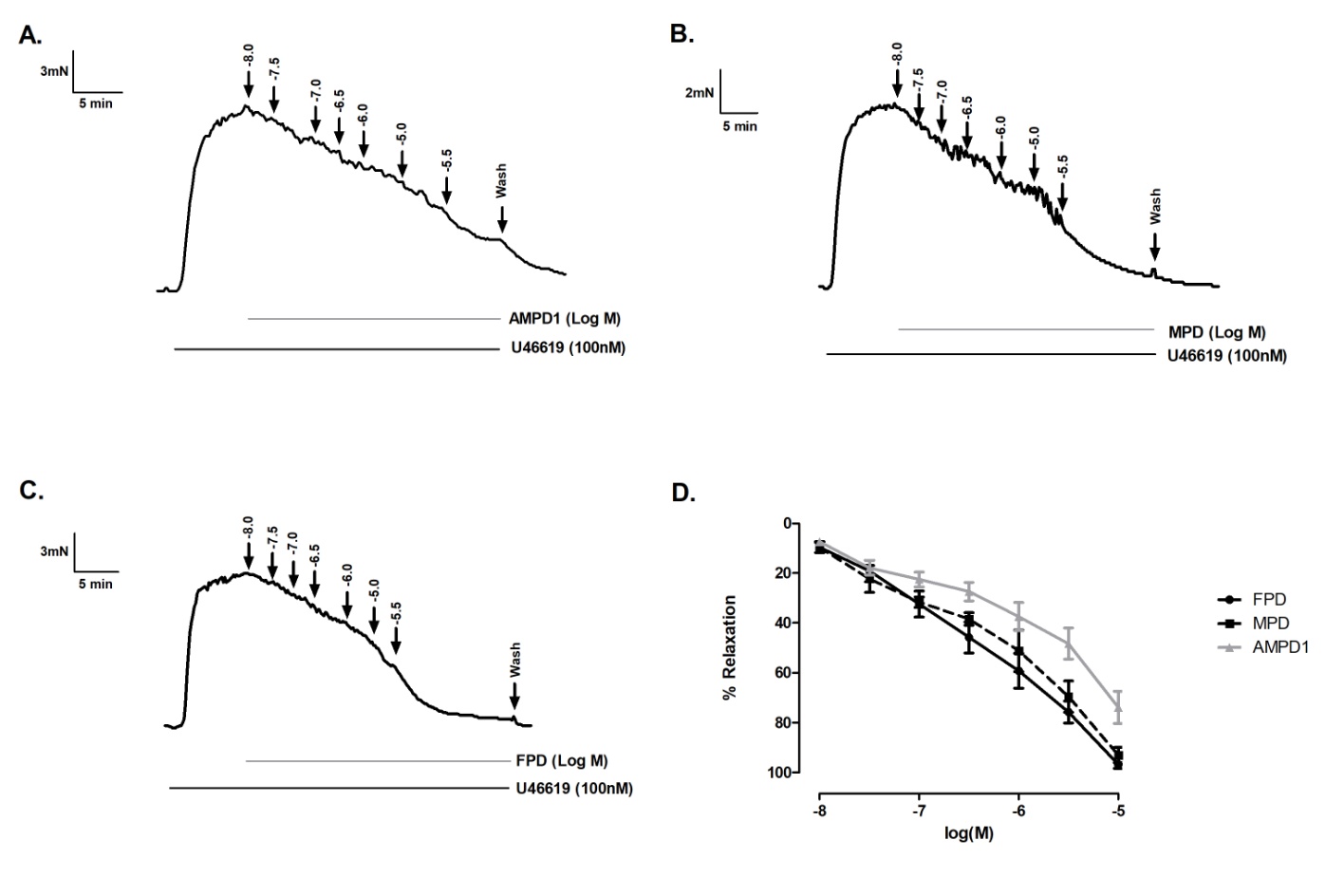
**

Figure S3: Homology 3D protein structure model (model_1) of sGC subunit β.

**
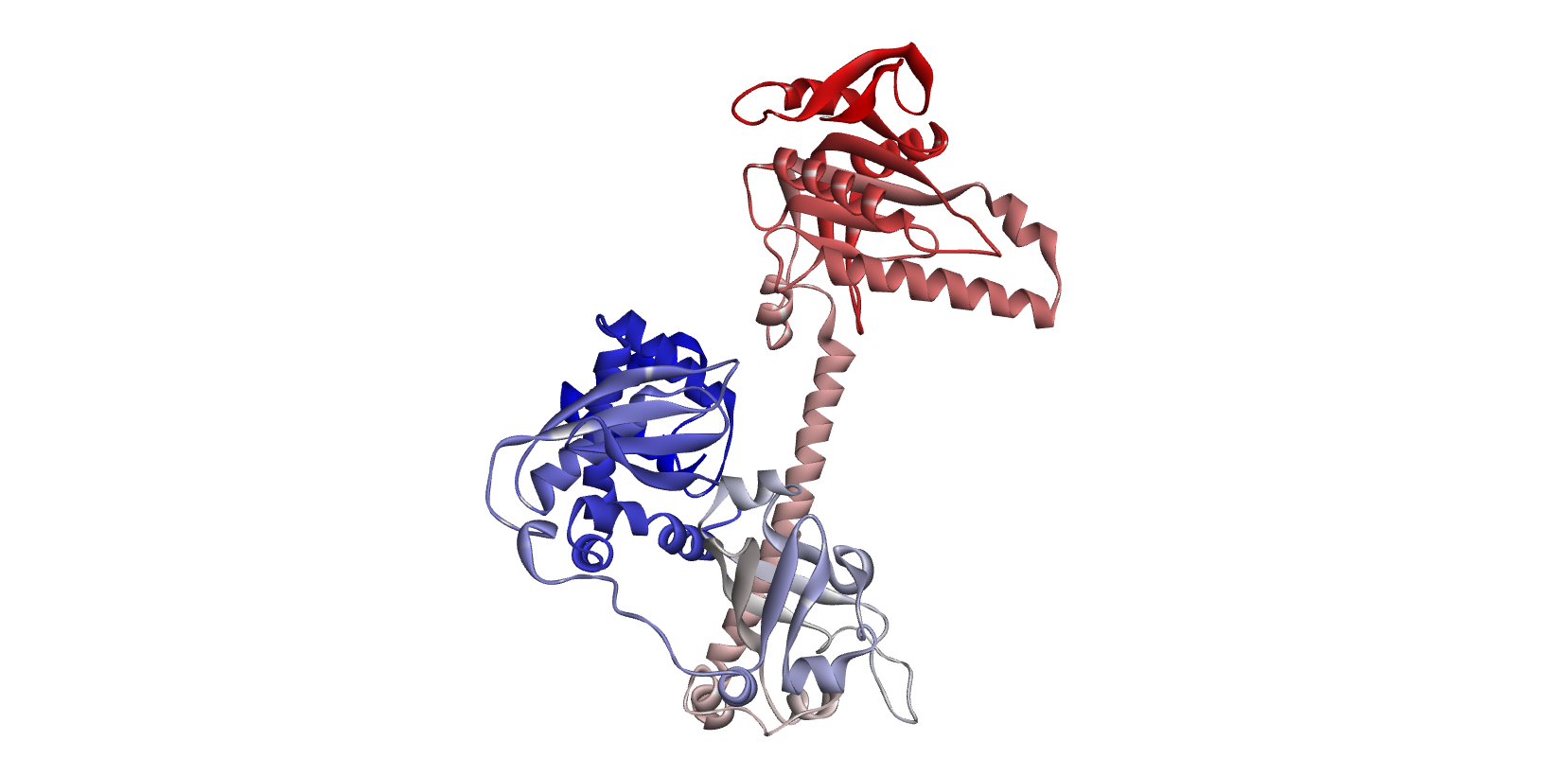
**
